# The role of statistical methods and artificial intelligence in inventory management for manufacturing industries: a systematic literature review

**DOI:** 10.3389/fdata.2026.1799073

**Published:** 2026-05-15

**Authors:** Arvia Dwi Royani, Mahfud Sholihin, Dewi Dewi, Novika Novika, Annisa Sorayya, Wahyu Nur Hanifah, Rizki Ramadhani Arif Trilana, Paolina Buton

**Affiliations:** 1 Department of Management, Faculty of Economics and Business, Universitas Gadjah Mada, Yogyakarta, Indonesia; 2 Department of Accounting, Faculty of Economics and Business, Universitas Gadjah Mada, Yogyakarta, Indonesia

**Keywords:** artificial intelligence, hybrid approach, inventory management, manufacturing industry, statistical methods

## Abstract

Inventory management is a critical business process that affects the operational efficiency and competitiveness of manufacturing companies. Inaccurate inventory decisions can result in significant financial losses for companies. Demand variability poses a challenge in determining inventory levels, requiring more sophisticated, flexible forecasting methods. This study was conducted to examine the roles of statistical methods and Artificial Intelligence (AI) in inventory decision-making in the manufacturing industry, analyze the conditions under which each method is suitable, and evaluate the potential of a hybrid approach integrating statistical methods and AI. This study uses the Systematic Literature Review method with the PRISMA 2020 framework to ensure research transparency and accuracy. This study identifies articles from reputable databases indexed in Scopus. The findings show a significant shift in inventory management research. In the last decade, AI technology has dominated the literature at 62.5%, while statistical methods account for 25%, and hybrid methods have begun to emerge but remain limited to 12.5%. Based on the review of selected papers, statistical methods have proven to remain effective for consistent historical data and stable demand patterns. Conversely, in dynamic operational environments with large-scale data and complex nonlinear patterns, AI technology is superior. This study also found that the hybrid approach has great potential to balance accuracy, interpretability, and decision support, although the relevant literature remains limited. The implementation of technology in the manufacturing industry faces several obstacles, including limited data quality, a skills gap in technology, and the black-box nature of complex AI. This review provides a systematic and critical synthesis of methodological patterns and operational fit in the use of statistical, AI, and hybrid methods for manufacturing inventory management. Future research is recommended to focus on the development of interpretable AI, modular hybrid frameworks, and the use of real industry data to ensure that academic innovations can be applied in the manufacturing industry.

## 1 Introduction

Inventory management is a crucial process in supply chain management, as it directly affects operational efficiency, costs, and customer satisfaction (Aburto and Weber, 2007; Ahmed et al., 2024; Albayrak Ünal et al., 2023; Jauhar et al., 2024; Tang et al., 2023). In this age of globalization, the ability to forecast demand and optimize inventory levels has become a crucial factor for business competitiveness and sustainability (Ahakonye et al., 2024; Danach and Dirani, 2024). Companies must be able to identify and capitalize on every available opportunity (Jauhar et al., 2024). Decisions about what, how much, and when to purchase raw materials or finished goods are highly dependent on the accuracy of inventory forecasting (Aburto and Weber, 2007). Mismanagement of inventory can lead to a mismatch between production and demand (Ahakonye et al., 2024; Ahmed et al., 2024). Overstock can lead to wasted storage and maintenance costs, as well as the risk of stock damage (Affonso et al., 2024; Ahakonye et al., 2024; Van Steenbergen and Mes, 2020; Walter et al., 2025). Furthermore, stockouts can also increase the risk of delivery delays and order fulfillment failures (Ben-Daya and Hassini, 2019). Therefore, improving inventory forecasting should be a priority for companies (Ben-Daya and Hassini, 2019; Danach and Dirani, 2024).

Statistical methods such as ARIMA models, exponential smoothing, and methods based on the normal distribution have long been used as the basis for inventory forecasting because they are relatively simple, transparent, and easy to implement in inventory policy making (Aburto and Weber, 2007; Kourentzes and Trapero, 2020; Spiliotis et al., 2022). However, several studies have shown that purely statistical methods are not sufficiently adaptive to rapid changes in demand patterns, leading more often to biased inventory estimates (Affonso et al., 2024; Kourentzes and Trapero, 2020; Tang et al., 2023). In conducting inventory forecasting, companies encounter various challenges, such as limited historical data, demand fluctuations driven by prices and consumer behavior, and changes in competitor strategies (Aburto and Weber, 2007; Van Steenbergen and Mes, 2020; Walter et al., 2025). Furthermore, from a broader perspective, small changes in consumer demand at the retail level can lead to major fluctuations at the wholesale, distributor, and manufacturer levels, known as the bullwhip effect (Aburto and Weber, 2007). This makes efficient inventory management more challenging (Aburto and Weber, 2007). Therefore, developing more advanced and adaptable forecasting methods is crucial to meet high demand variability.

The rise of the big data era has dramatically increased the amount of data produced by individuals, driving progress in Artificial Intelligence, particularly in machine learning, deep learning, and reinforcement learning (Liu et al., 2025). Artificial Intelligence (AI) is a computer system created to understand and imitate human behavior and intelligence (Salani and Tapfuma, 2025). It creates new opportunities to enhance the accuracy and reliability of demand forecasting in the industrial sector (Jauhar et al., 2024; Walter et al., 2025). AI technology can transform industrial processes, particularly in inventory management, from planning and scheduling to optimization (Ahakonye et al., 2024). In the early stages, AI mainly complemented statistical methods, especially for capturing nonlinear patterns that could not be modeled with ARIMA or exponential smoothing using neural network techniques (Aburto and Weber, 2007). However, the role of AI is now increasingly complex. AI-based approaches, such as random forests and quantile regression forests, can provide point estimates while quantifying demand uncertainty through interval estimates (Van Steenbergen and Mes, 2020).

However, the application of AI in inventory management has its limitations and challenges. Some challenges companies might encounter include limited internal technology expertise and a traditional business culture that prioritizes cost reduction over rapid innovation (Wu et al., 2025). In addition, implementing AI requires high-quality, clean, and relevant data (Gutierrez et al., 2025). Model interpretation and decision-making confidence are also challenges that need to be addressed, as many AI algorithms are black boxes and difficult to explain to operational managers (Goltsos et al., 2022; Walter et al., 2025). On the other hand, highly complex AI models also risk overfitting, especially when applied to fluctuating and unstable demand data (Spiliotis et al., 2022; Tang et al., 2023).

Therefore, recent literature increasingly emphasizes the application of hybrid models that combine the advantages of statistical methods with the adaptive capabilities of AI (Aburto and Weber, 2007; Affonso et al., 2024). Using a hybrid approach, companies can preserve the transparency and ease of implementation of statistical methods while also leveraging the capabilities of AI in capturing nonlinear patterns and handling high uncertainty (Affonso et al., 2024; Tang et al., 2023). Thus, the development of inventory forecasting methods will focus not only on enhancing prediction accuracy but also on improving the applicability and interpretability of the model to support managerial decision-making (Goltsos et al., 2022; Walter et al., 2025).

Several reviews have been conducted to examine the application of artificial intelligence in supply chain management, but few have focused exclusively on inventory forecasting (Toorajipour et al., 2021). Previous paper reviews have shown that AI models, like deep learning and machine learning, can enhance demand forecasting accuracy compared to traditional statistical methods (Albayrak Ünal et al., 2023; Walter et al., 2025). These reviews indicate that AI research still mainly focuses on forecasting, with little emphasis on using those forecasts to set safety stock, reorder points, service levels, or to address the bullwhip effect (Albayrak Ünal et al., 2023; Walter et al., 2025).

The main research gap this paper focuses on is the lack of a comprehensive study that thoroughly integrates traditional statistical methods with AI techniques to support decision-making in inventory management (Albayrak Ünal et al., 2023). Previous studies have tended to focus on the ability of AI to improve accuracy, but there has been little synthesis of how forecasting results using statistical and AI methods influence the risk of inventory overstocking and understocking (Toorajipour et al., 2021; Walter et al., 2025). Furthermore, the connection between demand uncertainty, limitations, and the strengths of each statistical and AI method in supporting inventory decision-making has not been examined systematically within an integrated review perspective. This gap presents an opportunity for a paper review on the role of statistics and AI in inventory management decisions within the manufacturing industry.

Based on this research gap, this study aims to evaluate the role of statistical and AI methods in supporting inventory management decisions in the manufacturing industry, and to identify the operational conditions under which AI methods outperform traditional statistical methods, and vice versa. Additionally, this study will discuss hybrid approaches that combine statistical and AI methods in inventory management. Further, this study will discuss the main obstacles to implementing statistical and AI methods in inventory decision-making that future research should address. Rather than merely summarizing trends, this review provides a systematic and critical synthesis of methodological patterns, operational fit, and unresolved gaps in the literature on manufacturing inventory management.

## 2 Materials and method

This study employed a structured approach, with specific methods and materials, to identify and classify relevant literature. The review primarily followed the PRISMA (Preferred Reporting Items for Systematic Reviews and Meta-Analyses) 2020 framework (Page et al., 2021). The PRISMA method has been widely acknowledged as the global standard for reporting systematic reviews and meta-analyses. This method offers the most recent guidelines for enhancing transparency and accuracy in conducting systematic reviews (Efendi and Shao, 2025). PRISMA is designed to ensure that the process of identifying, screening, evaluating, and including literature is conducted systematically and documented clearly. This helps reduce selection bias in review studies (Abyaneh et al., 2025; Baryannis et al., 2019).

The PRISMA method is applied in four stages (Efendi and Shao, 2025), namely (1) identifying research questions (RQ), (2) identifying paper sources, (3) searching for papers that match the RQ, and (4) analyzing the search results. The PRISMA method was selected over other approaches, such as narrative reviews, because it is recognized as the international standard in SLR research (Page et al., 2021). It has a systematic framework, offers transparency in research stages with clear paper selection and screening, reduces selection bias, provides flexibility for different types of studies, is compatible with modern databases, helps reviewers verify the validity of SLR, and emphasizes the quality of the included papers (Page et al., 2021).

### 2.1 Identifying the research questions (RQ)

This systematic review formulates critical questions to explore specific issues related to the roles of statistics and AI in inventory management. These questions primarily focus on:

RQ1: How have statistical methods and AI methods been used in inventory management decision-making in the manufacturing industry over the past decade?

RQ2: In which operational conditions are AI methods proven to outperform traditional statistical methods, and vice versa?

RQ3: How extensively does existing literature explore hybrid approaches that combine statistics and AI? What are the advantages of these methods, and what challenges do they encounter?

RQ4: What are the technical challenges, limitations, and recommendations for future research on optimizing the integration of statistical methods and AI in inventory management decision making?

### 2.2 Collecting and selecting relevant articles according to the research questions

Research on the application of statistics and AI in supply chain management is already extensive from reputable sources. However, research specifically targeting inventory management within the manufacturing industry remains notably limited. The primary objective of this study is to analyze research journals indexed in Scopus. The database search was conducted on January 2026, covering publications from 2015 to 2025. All articles must be accessible in full text and written in the English language. To ensure a comprehensive identification of relevant literature, article searches were conducted via publisher websites without supplementary hand-searching of reference lists as well as broader academic databases, such as ScienceDirect and Google Scholar, employing consistent keyword combinations. The results obtained from ScienceDirect and Google Scholar substantially overlapped with those gathered through publisher websites. Articles that met the inclusion criteria across all search channels demonstrated consistency, thereby confirming that the final selection was robust and not sensitive to the choice of search interface. Given this overlap, the deliberate focus on journals indexed in Scopus reflects a quality-oriented inclusion criterion, guaranteeing that all examined articles have undergone rigorous peer review in accordance with international academic standards.

Articles meeting these criteria were selected using the inclusion and exclusion methods. The final dataset consists of publications that meet all inclusion criteria. Articles matching these keywords will be reviewed further. Moreover, the selection criteria shown in Table 1 clarify the inclusion and exclusion parameters for these articles. The keyword set is as follows:

**Table 1:** Inclusion and exclusion.

Inclusion	Exclusion
Paper published in 2015-2025	Paper published outside 2015-2025
Full text paper	The paper does not include the full text
Paper written in English	Paper written in a language other than English
Papers published in Scopus indexed Journals	Articles not published in Scopus-indexed journals
Research Papers	Books, reviews, short articles, magazine articles, proceedings
Papers addressing inventory management in the manufacturing industry using statistical methods, AI, or both	Paper not related to inventory management in manufacturing using statistical methods, AI, or both.

TITLE-ABS-KEY (“inventory management”) AND (“demand forecasting” OR “inventory optimization”) AND (“artificial intelligence” OR “machine learning” OR “deep learning” OR “reinforcement learning” OR “ARIMA” OR “moving average” OR “statistical method”) AND (“manufacturing”).

The study selection process was conducted in several stages to reduce selection bias and improve transparency. First, records retrieved from the search were filtered by publication year, language, document type, and database scope. Second, duplicate records were removed. Third, titles and abstracts were screened for topical relevance to inventory management in the manufacturing industry using statistical methods, AI, or hybrid approaches. At this stage, articles were excluded if they focused on broader supply chain topics without a clear inventory management component, addressed non-manufacturing sectors, or discussed forecasting techniques without linking them to inventory-related decisions. Fourth, the remaining articles were assessed through full-text review to confirm their fit with the predefined inclusion criteria. Eligibility decisions were discussed among the authors whenever uncertainty arose, and final inclusion was determined through consensus.

**Figure 1:**
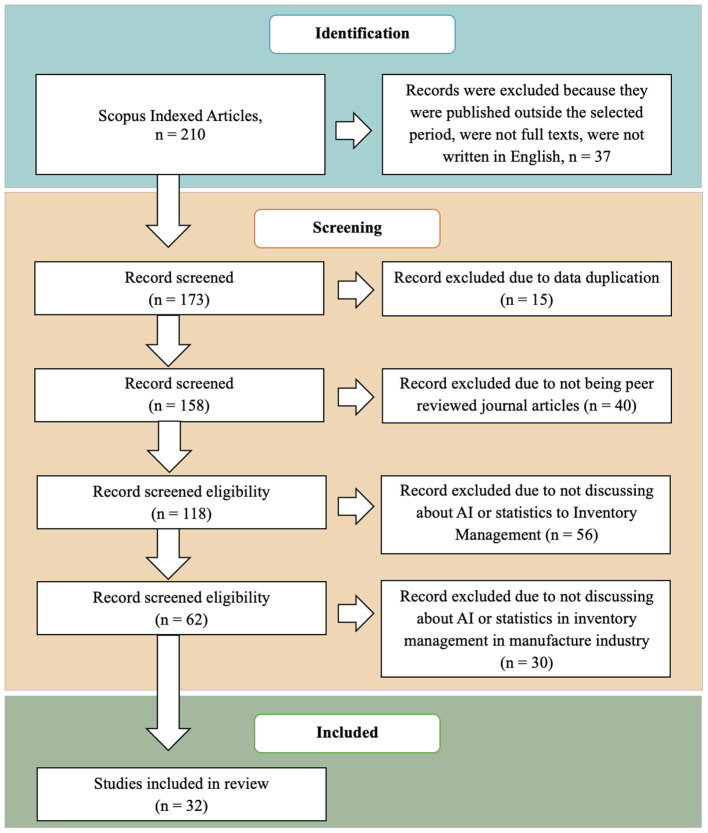
Flowchart of the article selection process.

**Figure 2:**
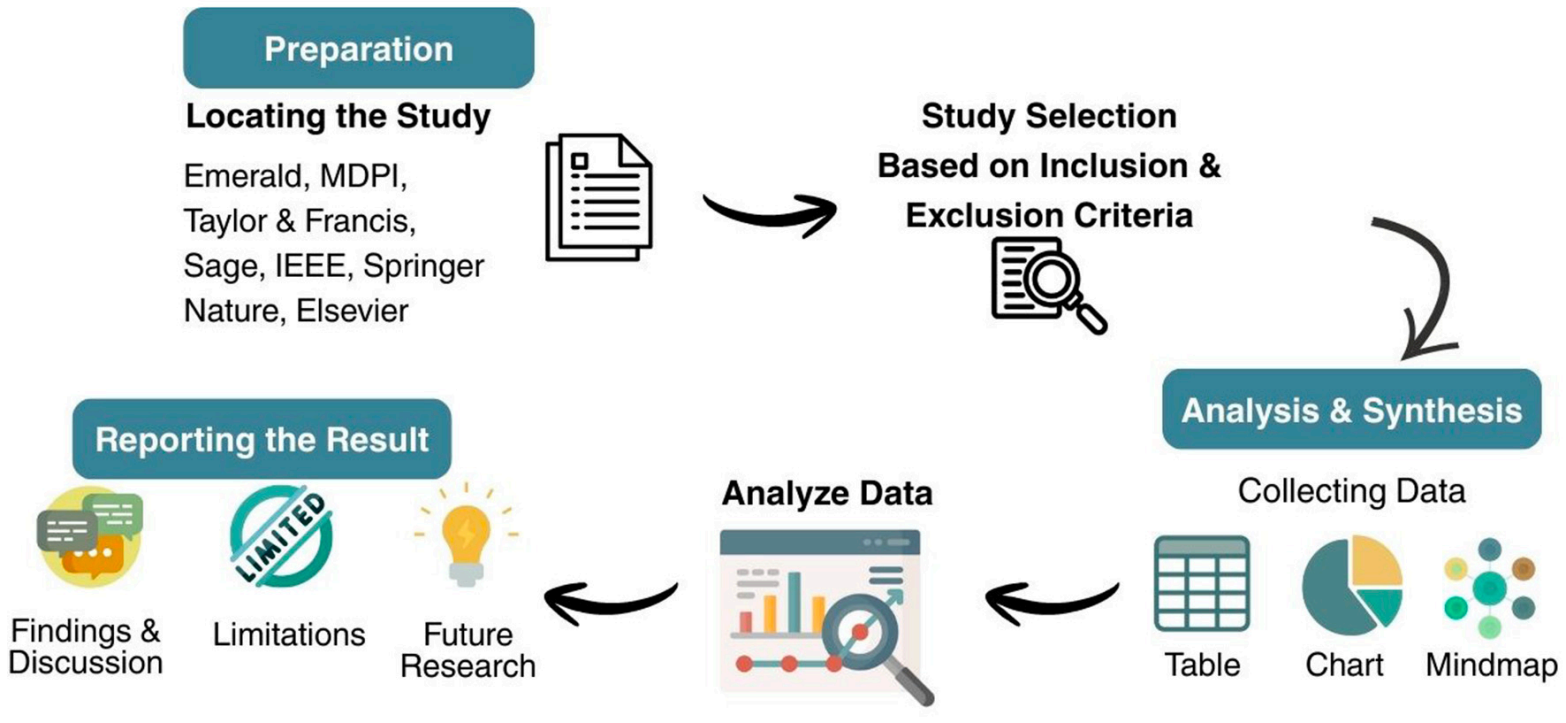
Research process of systematic literature review with PRISMA method.

Researchers identified 210 articles published in Scopus-indexed journals by using specific categories and keyword searches. The researchers then selected only publications from 2015 to 2025, resulting in 173 articles that met these time constraints and were written in English. After screening, the researchers identified 15 duplicate articles and removed them, leaving 158 articles. After excluding several non-journal articles and proceedings, 118 articles remained for further analysis. Then, after evaluating relevance and reviewing each abstract of articles to assess their suitability for inventory management, 62 relevant articles were identified. After this screening, the researchers conducted a detailed review of the industry scope. The study focused on the manufacturing industry. After a thorough evaluation, 32 articles were selected for further analysis and moved on to the review phase. Figure 1 shows how articles were included or excluded, and Figure 2 illustrates the research process conducted using the PRISMA method. Additionally, Table 2 presents a detailed overview of these articles, including references, publishers, publication dates, methods, and citations, offering a comprehensive view of the reviewed literature.

**Table 2:** Selected paper.

No	References and publication date	Publisher	Scopus index	Citation
1.	Fattah et al. (2018)	Sage	Q2	286
2.	Jaipuria and Mahapatra (2019)	Emerald	Q2	11
3.	Hajek and Abedin (2020)	IEEE	Q1	89
4.	Kourentzes and Trapero (2020)	Elsevier	Q1	71
5.	Salinas et al. (2020)	Elsevier	Q1	1,915
6.	Tliche and Taghipour (2020)	Elsevier	Q1	36
7.	Gammelli et al. (2022)	Elsevier	Q1	39
8.	Kim et al. (2022)	MDPI	Q2	16
9.	Preil and Krapp (2022)	Springer	Q1	67
10.	Spiliotis et al. (2022)	Springer	Q2	92
11.	Li et al. (2023)	Taylor & Francis	Q1	13
12.	Wahedi et al. (2023)	MDPI	Q2	8
13.	Affonso et al. (2024)	Elsevier	Q1	9
14.	Ahakonye et al. (2024)	Elsevier	Q1	11
15.	Bo and Xu (2024)	IEEE	Q1	14
16.	Danach and Dirani (2024)	IEEE	Q1	38
17.	Jackson and Jesus Saenz (2024)	Taylor & Francis	Q1	81
18.	Jauhar et al. (2024)	Taylor & Francis	Q1	68
19.	Kumar et al. (2024)	Elsevier	Q2	15
20.	Pasupuleti et al. (2024)	MDPI	Q2	112
21.	Sagaert and Kourentzes (2024)	Elsevier	Q1	1
22.	Singh and Mishra (2024a)	Springer	Q1	18
23.	Singh and Mishra (2024b)	Taylor & Francis	Q1	13
24.	Sukolkit et al. (2024)	Elsevier	Q1	5
25.	Aiello et al. (2025)	Elsevier	Q1	1
26.	Arvind et al. (2025)	IEEE	Q1	0
27.	Karpagavigneswari and Kamali (2025)	Springer	Q2	0
28.	Kumar and Khedlekar (2026)	Springer	Q2	0
29.	Liu et al. (2025)	Sage	Q1	13
30.	Raouf et al. (2025)	Elsevier	Q1	0
31.	Sandra et al. (2025)	Springer	Q2	6
32.	Shahin et al. (2025)	Springer	Q1	9

### 2.3 Quality assessment

We assess the selected studies based on defined quality criteria to increase reability of the results. We use the guidelines suggested by Kitchenham et al. (2015) for performing rigorous quality assessment of the selected papers. To assess the selected articles, the quality criteria checklist contains the following questions:Q1: Is there a clear statement defining the objective and aim of the research?Q2: Is there an adequate description justifying the choice of research area?Q3: Is the research method appropriate to address the aims of the research?Q4: How feasible is the method followed to achieve results?Q5: Is there clear and coherent reporting of the findings?Q6: Is the study of value for research and practice?

If descriptions are unclear, the paper receives a score of 0 = No. If there is only a partial description, the score is 0.5 = Partial. Otherwise, the score is 1 = Yes. We discuss the scores assigned to each of the studies in this review in Section 3.1. Quality assessment was conducted by two independent reviewers.

The quality assessment was included not only to document the methodological adequacy of the selected studies, but also to support the interpretation of the synthesis. The checklist adapted from Kitchenham et al. (2015) was considered appropriate for this review because the included studies varied in analytical design, modeling approach, and practical orientation, making a flexible and broadly applicable appraisal tool more suitable than a design-specific instrument. The six criteria were used to assess whether each study clearly stated its objectives, justified the research context, applied an appropriate and feasible method, reported findings coherently, and provided value for research or practice. Rather than using the scores as a basis for excluding studies, the assessment was used to evaluate the overall robustness of the evidence base and to interpret the strength and limitations of the conclusions drawn from the synthesis.

## 3 Analysis and synthesis

### 3.1 Quality assessment result

The quality of the 32 selected studies was assessed using a checklist of six criteria (QC1–QC6) as defined in Section 2.3, following the guidelines proposed by Kitchenham et al. (2015). Each criterion was scored 1 (Yes), 0.5 (Partial), or 0 (No), yielding a maximum total score of 6 per study. The results in Table 3 indicate that most selected studies achieved high quality scores. Thirty studies clearly defined their research objectives (QC1), provided adequate justification for the chosen research area (QC2), employed appropriate research methods (QC3), applied feasible methods to achieve results (QC4), reported findings clearly and coherently (QC5), and demonstrated clear value for both research and practice (QC6). Four studies received slightly lower scores due to partial satisfaction of two criteria, and six studies scored 5.5 of 6.

**Table 3:** Quality assessment of selected papers.

No	References	QC1	QC2	QC3	QC4	QC5	QC6	Score
1.	Fattah et al. (2018)	1	0.5	1	1	1	0.5	5
2.	Jaipuria and Mahapatra (2019)	1	1	1	1	1	1	6
3.	Hajek and Abedin (2020)	1	1	1	1	1	1	6
4.	Kourentzes and Trapero (2020)	1	1	1	1	1	1	6
5.	Salinas et al. (2020)	1	1	1	1	1	1	6
6.	Tliche and Taghipour (2020)	1	1	1	1	1	1	6
7.	Gammelli et al. (2022)	1	1	1	1	1	1	6
8.	Kim et al. (2022)	1	1	1	1	1	1	6
9.	Preil and Krapp (2022)	1	1	1	1	1	1	6
10.	Spiliotis et al. (2022)	1	1	1	1	1	1	6
11.	Li et al. (2023)	1	1	1	1	1	1	6
12.	Wahedi et al. (2023)	1	1	1	1	1	1	6
13.	Affonso et al. (2024)	1	1	1	1	1	1	6
14.	Ahakonye et al. (2024)	1	1	1	1	1	0.5	5.5
15.	Bo and Xu (2024)	1	0.5	1	1	1	0.5	5
16.	Danach and Dirani (2024)	1	1	1	0.5	1	0.5	5
17.	Jackson and Jesus Saenz (2024)	1	1	1	0.5	1	1	5.5
18.	Jauhar et al. (2024)	1	1	1	1	1	1	6
19.	Kumar et al. (2024)	1	1	1	0.5	1	1	5.5
20.	Pasupuleti et al. (2024)	1	1	1	0.5	1	0.5	5
21.	Sagaert and Kourentzes (2024)	1	1	1	1	1	1	6
22.	Singh and Mishra (2024a)	1	1	1	1	1	1	6
23.	Singh and Mishra (2024b)	1	1	1	1	1	1	6
24.	Sukolkit et al. (2024)	1	1	1	1	1	1	6
25.	Aiello et al. (2025)	1	1	1	1	1	1	6
26.	Arvind et al. (2025)	1	1	1	0.5	1	1	5.5
27.	Karpagavigneswari and Kamali (2025)	1	1	1	1	1	0.5	5.5
28.	Kumar and Khedlekar (2026)	1	1	1	0.5	1	1	5.5
29.	Liu et al. (2025)	1	1	1	1	1	1	6
30.	Raouf et al. (2025)	1	1	1	1	1	1	6
31.	Sandra et al. (2025)	1	1	1	1	1	1	6
32.	Shahin et al. (2025)	1	1	1	1	1	1	6

Overall, the quality assessment indicates that the selected studies range from satisfactory to high quality, with an average score of around 5.8 out of 6. No study received a score below 5, which underscores the reliability and rigor of the literature selected for this systematic review. The results of the quality assessment also carry significant implications for the synthesis. Given that no study scored below 5 and the average score was approximately 5.8 out of 6, the overall evidence base can be regarded as methodologically sufficient for a narrative synthesis of trends, methodological roles, and implementation challenges. Nonetheless, these scores should not be interpreted as indicative that all findings possess equal strength. Several studies attained slightly lower scores due to partial justification of the research setting, limited discussion of feasibility, or weaker articulation of practical value. Consequently, conclusions concerning the broad shift from statistical methods to AI are relatively well supported across the sample, while conclusions regarding the superiority or practical efficacy of specific hybrid configurations remain more tentative owing to the limited number of such studies and the uneven depth of reporting among them.

### 3.2 Synthesis

**Figure 3:**
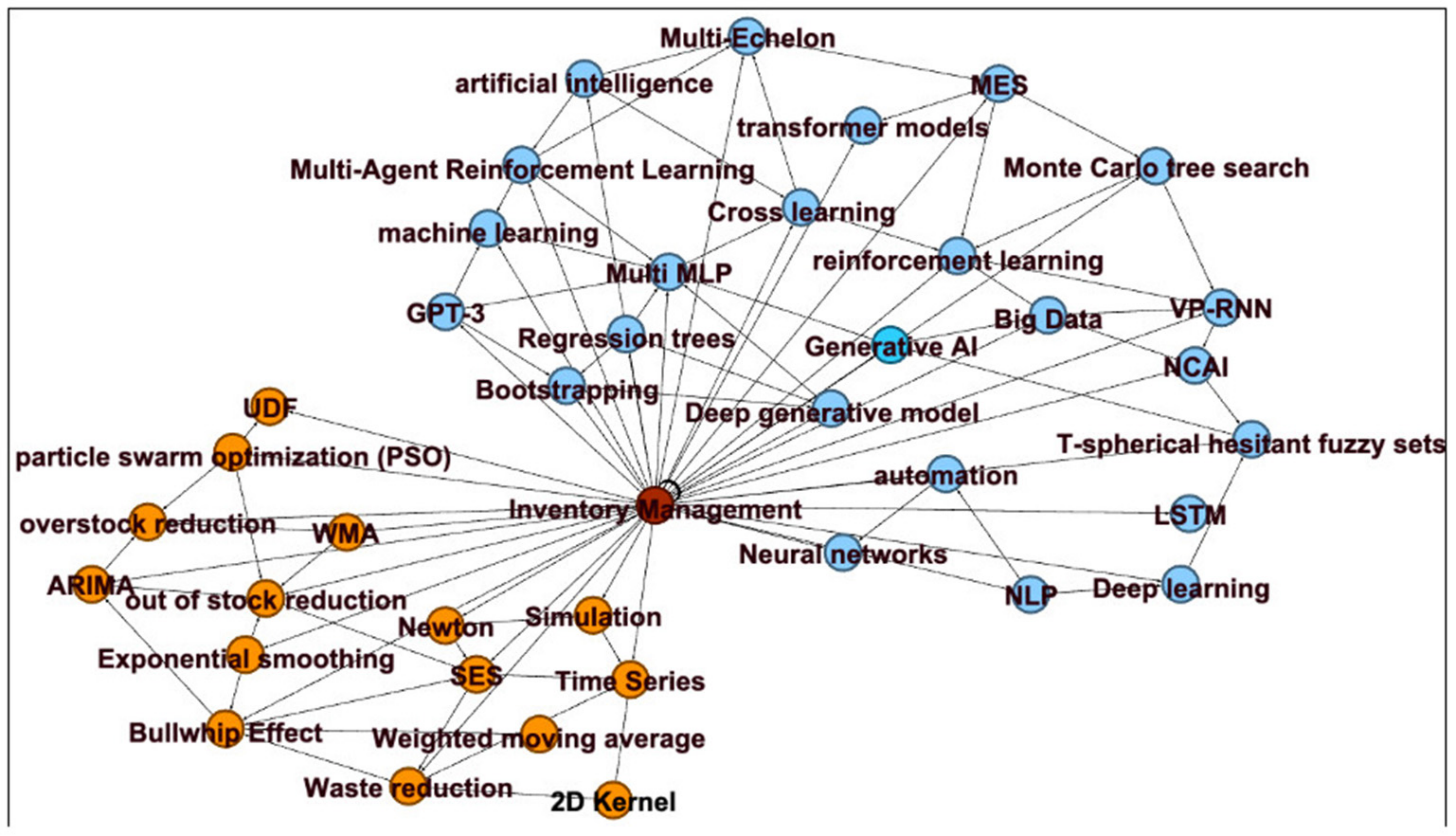
Keyword mapping derived from a systematic process.

**Figure 4:**
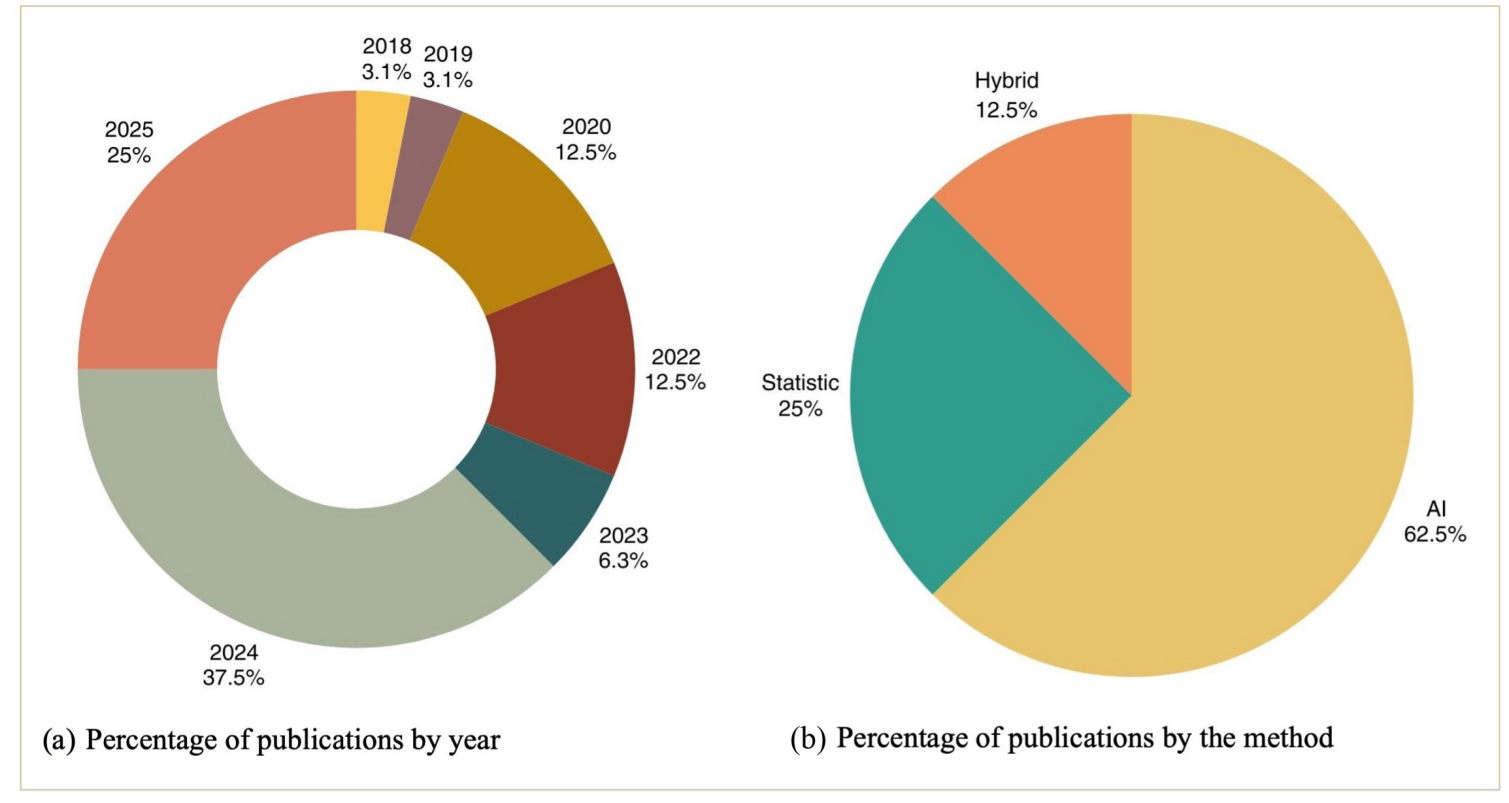
Descriptive statistical analysis of literature collection based on **(a)** year of publication, and **(b)** method.

The dataset was extracted from the 32 selected articles and processed with Gephi to generate a keyword network that reveals connections among the chosen articles. This visual analysis highlights current research trends and identifies potential gaps for future studies. Figure 3 illustrates the keyword co-occurrence network generated from the 32 selected studies using Gephi. Nodes represent keywords extracted from the selected articles, and edges indicate co-occurrence relationships between keywords within the same study. The network is color-coded to distinguish two dominant methodological streams, orange nodes denote keywords associated with statistical methods, while blue nodes signify keywords linked to AI-based methods. The relatively sparse connections between the orange and blue clusters visually corroborate the research gap identified in this review, integrated frameworks that systematically combine statistical and AI methods remain underdeveloped in the literature. The denser expansion of the blue cluster compared to the orange cluster further reflects the quantitative shift observed in the publication trend analysis (Figure 4), wherein AI-based studies now constitute 62.5% of the reviewed literature.

**Figure 5:**
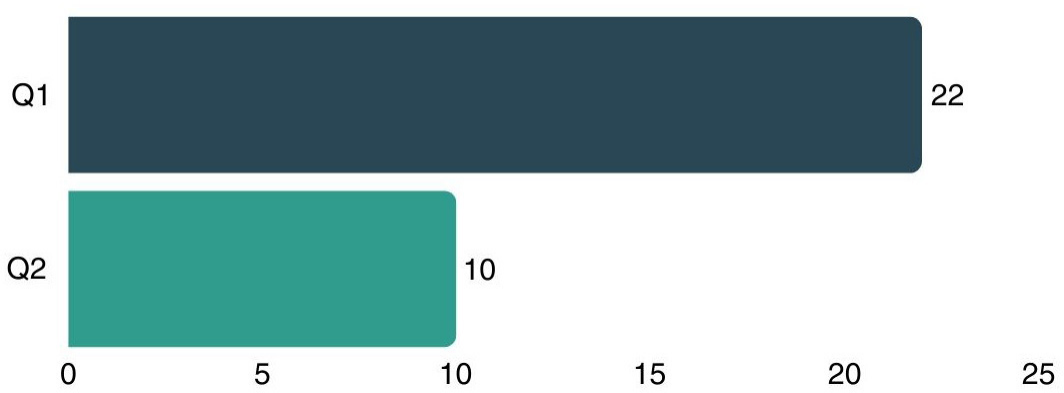
Articles by quartile.

After collecting various relevant articles, the next step is to analyze and synthesize the data. This aims to describe the overall relationships and connections and identify the relationships between different studies (Toorajipour et al., 2021). The articles collected were published from 2015 to 2025. However, since none of the articles published in 2015, 2016, 2017, and 2021 met all the inclusion criteria, the selected articles were those published between 2018 and 2025, excluding 2021. Figure 5 shows that all selected articles come from reputable journals, with 22 articles from Scopus Q1-indexed journals and the remaining 10 from Q2 journals.

Publication on the application of statistics and AI in inventory management within the manufacturing industry continues to increase. Details of publications based on those year are: 2018 (3.1%), 2019 (3.1%), 2020 (12.5%), 2022 (12.5%), 2023 (6.3%), 2024 (37.5%), and 2025 (25%). Furthermore, this paper categorizes articles based on the methods used for inventory management, namely articles dominated by AI applications (62.5%), followed by statistics (25%), and hybrid methods (12.5%). Hybrid methods are strategies that combine statistical and AI techniques within a single framework. Therefore, articles discussing both statistics and AI solely for comparison are not considered hybrid methods. Figure 4 shows the results of categorizing articles by publication year and methodology. Figure 4a shows the percentage of selected articles by publication year. Meanwhile, Figure 4b shows the distribution of articles by inventory management method.

**Figure 6:**
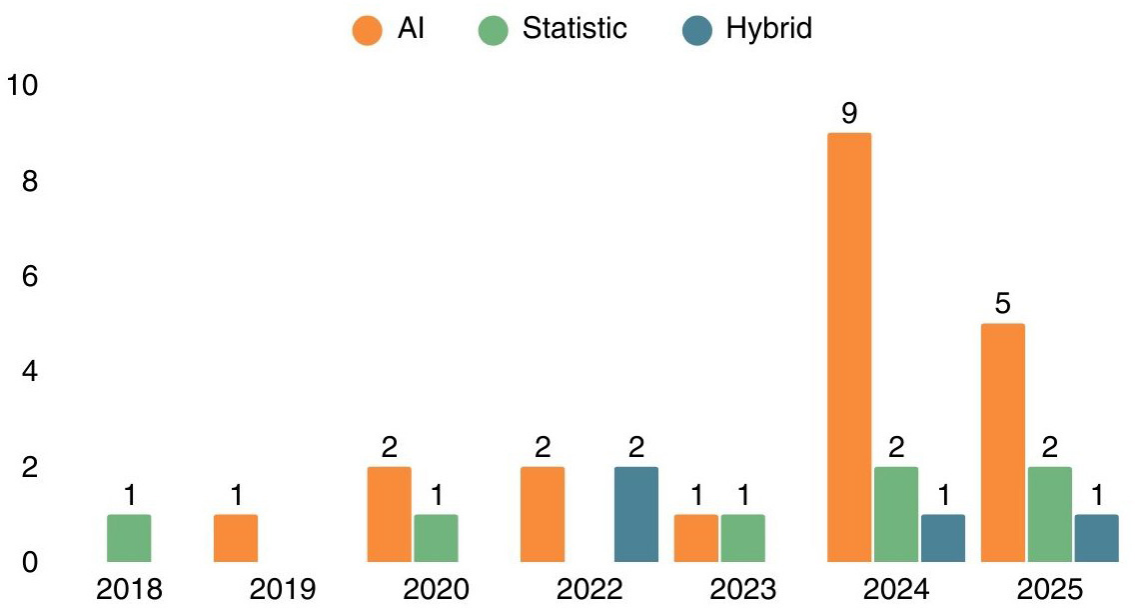
Number of publications by year and method used for inventory management.

Based on a synthesis of selected articles, Figure 6 shows a shift in the methods used in inventory management research over the past 8 years. Early studies remained focused on classical statistical methods that emphasized demand time series modeling and the assessment of prediction accuracy. As 2019 began, literature on AI in inventory management started to emerge but remained limited. Then, in line with the increasing availability of data, computing power, and industry demand for more adaptive inventory systems, the application of AI surged until 2025. Articles discussing AI appeared most frequently in 2024. The separate application of statistics and AI proved to have limitations. Statistics and Artificial Intelligence (AI) can be used together to compensate for both of their weaknesses. Thus, research using both statistical methods and AI in inventory management started appearing in 2022, but was limited. The latest literature is dominated by AI-based methods, while hybrid methods serve as a response to the limitations of single-technique methods.

**Figure 7:**
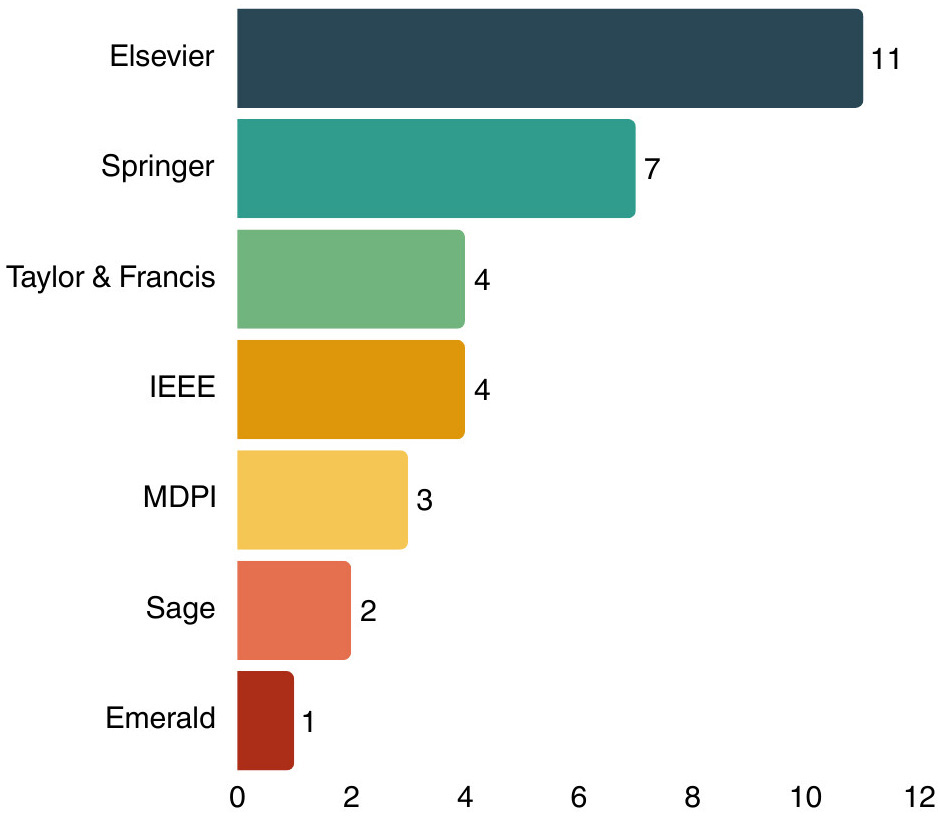
Number of publications by publisher type.

In Figure 7 shows that among all articles meeting the inclusion criteria, 34.38% were published by Elsevier, and 21.88% by Springer. Next, Taylor & Francis and IEEE publishers each represented 12.5%. The remaining articles were from MDPI, Sage, and Emerald publishers, representing 9.37, 6.25, and 3.12%, respectively. Looking at the data more closely, the articles originated from 27 different journals, with the majority from IEEE Access (4 articles), Taylor & Francis International Journal of Production Research (3 articles), Springer Annals of Operations Research (2 articles), MDPI Applied Sciences (2 articles), and the remaining 23 journals each contributing one article.

Selected articles have a significant impact, as shown by the number of citations. The most influential article is the one by Salinas et al. (2020), published in the International Journal of Forecasting with 1,915 citations. Next are the articles by Fattah et al. (2018), which has been cited 286 times, and the article by Pasupuleti et al. (2024), which has been cited 112 times. This indicates that the selected articles are from reputable sources and have undergone thorough peer review.

## 4 Results and discussion

RQ1: How have statistical methods and AI methods been used in inventory management decision-making in the manufacturing industry over the past decade?

The classification of studies in Tables 4–6 follows a structured coding scheme developed by the authors based on the research questions. Decision support complexity refers to whether the method proposed in the study supports only predictive tasks (low complexity) or extends to prescriptive decisions such as reorder point, safety stock, or inventory optimization (high complexity). Reported effectiveness is coded based on the authors' own evaluation as stated in each study. Methodological typologies (Statistical, AI, Hybrid) were assigned based on the previous studies, namely the studies of Sarker (2021) and Seyedan and Mafakheri (2020).

**Table 4:** Article with the highest number of citations.

No	References	Source publisher	Source journal	Citation
1.	Salinas et al. (2020)	Elsevier	International Journal of Forecasting	1,915
2.	Fattah et al. (2018)	Sage	International Journal of Engineering Business Management	286
3.	Pasupuleti et al. (2024)	MDPI	Logistics	112
4.	Spiliotis et al. (2022)	Springer	Operational Research	92
5.	Hajek and Abedin (2020)	IEEE	IEEE Access	89
6	Jackson and Jesus Saenz (2024)	Taylor & Francis	International Journal of Production Research	81
7.	Kourentzes and Trapero (2020)	Elsevier	International Journal of Production Economics	71
8.	Jauhar et al. (2024)	Taylor & Francis	International Journal of Production Research	68
9.	Preil and Krapp (2022)	Springer	Annals of Operations Research	67
10.	Gammelli et al. (2022)	Elsevier	Transportation Research Part C: Emerging Technologies	39
11.	Danach and Dirani (2024)	IEEE	IEEE Access	38
12.	Tliche and Taghipour (2020)	Elsevier	European Journal of Operational Research	36

**Table 5:** An overview of statistics role.

References	Methods	Type	Task	Limitation
Affonso et al. (2024)	Heuristic Method	Bootstrap WSS, B2-OPT, BSWAP	To forecast with historical data and find quite a good solution	Cannot handle highly complex data
(Aiello et al. 2025)	optimization method	Closed-loop newsvendor model (CLSC)	To manage inventory within a closed-loop supply chain	Not hold for fresh products with variable shelf lives
(Fattah et al. 2018)	Time Series Analysis	ARIMA	To model and predict future demand based on historical data	Limited forecast accuracy
(Karpagavigneswari and Kamali 2025)	optimization method	Red Panda Optimizer with Logistic Mapping (RPO-LM)	To optimizing inventory decisions	Assuming infinite and perfect product quality
(Kourentzes and Trapero 2020)	Time Series Analysis	Level exponential Smoothing	To generate estimates of expected demand	Just focus on forecast accuracy
(Kumar et al. 2024)	Time Series Analysis	Simple Exponential Smoothing (SES)	To forecast by leveraging historical data	Limited capturing intricate market dynamics
(Li et al. 2023)	Time Series Analysis	Naïve, sNaive, SES, MA, ARIMA, ETS, CRO, optCro, SBA, TSB, ADIDA, IMAPA, SA, Median, FFORMA, FIDE, DIVIDE	To predict demand pattern	Lacking an automatic procedure for choosing features
(Tliche and Taghipour 2020)	Time Series Analysis	WMA and Newton	WMA to forecast and Newton to minimize errors	Limited optimal weighting information

**Table 6:** An overview of AI roles.

References	Methods	Algoritm	Task	Limitation
(Ahakonye et al. 2024)	DL	LightGBM	To estimate materials inventory	Availability of raw inventory datasets
(Arvind et al. 2025)	SL	Xgboost, Random Forest (RF) classifier	To demand forecasting and predictive analytics	Need integration with advanced technology
Bo and Xu (2024)	OA	Particle Swarm Optimization (PSO)	Optimize inventory parameters	Not effectively adapt to the growing complexity
(Danach and Dirani 2024)	SL	Decision tree, RF, gradient boosting	To predict outcomes and optimize processes	Perlu integrasi dengan advanced technologies
(Hajek and Abedin 2020)	SL	RF, Multi Layer Neural Network (NN), Decision Tree, k-Nearest Neighbor, and Support Vector Machine (SVM)	To perform classification and prediction.	Assumption of static inventory policies and stationary customer demand
(Jackson and Jesus Saenz 2024)	NLP	GPT 3 -Codex	To perform simulations with Python	Limited studies
(Jaipuria and Mahapatra 2019)	GP	DWT-MGGP	To forecast by filtering data with DWT and processing with MGGP	Limit to one behavior of bullwhip effect
(Jauhar et al. 2024)	H	No-Code Artificial Intelligence (NCAI)	To predict without programming skills	Only focus on the prediction process
(Liu et al. 2025)	RL	HAPPO	To optimized inventory policies and reduced cost.	Limited information sharing
(Pasupuleti et al. 2024)	SL	Long Short-Term Memory (LSTM), CNNs	To prediction time series data	Fail to respond swiftly to unexpected market shifts
(Preil and Krapp 2022)	RL	Monte Carlo Tree Search (MCTS),	To forecast with multiple demand scenarios	Not implement in limited data
(Raouf et al. 2025)	SL	AI-based optimization framework	To transform stock into usable inventory	Lack of External Factor Integration
(Singh and Mishra 2024a)	SL	Decision Tree Classifier	To classify data by creating a series of decision rules	Limited exploration with advanced forecasting methods
(Singh and Mishra 2024b)	SL	Decision Tree Classifier	To forecast seasonal demand	Not combining machine learning for imperfect products
(Salinas et al. 2020)	DL	RNN	To forecast sequential data	Not joint prediction of multiple time series
(Sandra et al. 2025)	SL	Fuzzy multi criteria	To select advanced predictive analytics	Need expert judgement
(Shahin et al. 2025)	SL	Extreme Learning Machines (ELM), Classification and Regression Trees (CART), Gaussian Processes (GPR), KNN, Multilayer Perceptron (MLP), Support Vector Regression (SVR), XGB, LSTM	To enhance forecasting accuracy	The existence of limitations in the use of n lag
	DL	Bayesian Neural Network (BNN), Fully Convolutional Networks (FCNs), Generalized Regression Neural Network (GRNN)	To enhance forecasting accuracy	Data Requirements
(Spiliotis et al. 2022)	DL	Multi Layer Perceptron (MLP), BNN	To learn data relationships and make predictions	Not clear why some ML models perform better than other ML methods
	SL	RF, GBT (Gradient Boosted Trees), K-Nearest, SVR (Support Vector Regression), GP (Gaussian Process)	To predict daily Stock Keeping Unit (SKU)	Require large sample to produce effective predictions
(Sukolkit et al. 2024)	SL	LSTM (CNN, RNN, GRU)	To predict with noisy or changing data	Limited on univariate time series data
(Wahedi et al. 2023)	DL	Artificial Neural Network (ANN)	To forecast specific data	Lacks a universal method for monitoring overall forecast accuracy performance.

DL, deep learning; SL, supervised learning; OA, optimization algorithm; NLP, natural language processing; GP, genetic programming; H, heuristic; RL, reinforcement learning

Among the selected papers, various statistical and AI methods were used in inventory management within the manufacturing industry. We found that the most commonly used statistical method in inventory management within the manufacturing industry is time series analysis, which was used in 62.5% of the total articles applying statistical methods in inventory management. Time series analysis is the most widely used method due to its simple implementation, as most managers already understand how it works, and it can also be applied to seasonal and non-stationary data (Kumar and Khedlekar, 2026; Li et al., 2023). Kourentzes and Trapero (2020) emphasize that this time-series analysis method yields a small Mean Squared Error (MSE) at a low cost.

It is also supported by a study from Aburto and Weber (2007), which states that the time series method is more explainable and understandable because with this method we can understand the cause and effect relation in the data, as the model is transparent. Although time series has various advantages, it cannot be denied that this method also has limitations, as shown in our findings in Table 5. This method often provides limited predictive accuracy because it cannot capture complexities and dynamics in the market (Kumar et al., 2024) and lacks an automated procedure for selecting the best features or model parameters (Li et al., 2023). This is in line with research conducted by (Lv 2024), which researchers integrated time series and machine learning methods for supply chain optimization. Time series is known for its simplicity and effective predictive capabilities for stable and seasonal data trends, while machine learning methods are used for large data sets that require efficient computational performance. In addition to time series methods, there are also optimization and heuristic methods. While time series use past data to forecast the present, heuristic methods generate solutions that are good enough, without any guarantee of optimality (Affonso et al., 2024). Meanwhile, optimization methods find optimal solutions by maximizing or minimizing a specific objective function (Aiello et al., 2025; Karpagavigneswari and Kamali, 2025).

Based on 20 selected articles that apply AI methods in inventory management, 47.62% of articles used supervised learning methods. Supervised learning is a fundamental approach in machine learning that involves training algorithms with labeled datasets (Zafar et al., 2024). Most articles use different algorithms, such as random forest classifier, LSTM, decision tree classifier, Bayesian Neural Network (BNN), Support Vector Regression (SVR), and so on. LSTM can improve planning accuracy (Wang et al., 2025). Random forest classifiers, SVR, and decision tree classifiers are widely used for tabular data because they are capable of handling non-linear relationships and complex preprocessing, and they are easier to explain to practitioners in the manufacturing industry (Arvind et al., 2025; Danach and Dirani, 2024; Hajek and Abedin, 2020; Spiliotis et al., 2022). Supervised learning is most widely used because it is easier to implement than deep learning and is more suitable for structured data in the manufacturing industry, such as transaction data, inventory, prices, and other operational parameters (Arvind et al., 2025; Danach and Dirani, 2024; Raouf et al., 2025).

This aligns with research conducted by Xu and Bo (2025), which found that supervised learning is an intelligent prediction tool for forecasting demand and identifying issues in the supply chain disruptions. Research by Tatarczak (2024) found that AI techniques in Supply Chain Management has yielded valuable insights. However, this model is often highly dependent on the availability of high-quality, real-time historical data (Xu and Bo, 2025). Then, aside from supervised learning, 23.81% of the 20 articles used deep learning methods. This model can not only capture structured data, but also unstructured data such as images (Ahakonye et al., 2024; Salinas et al., 2020; Wahedi et al., 2023). Moreover, this model is highly reliable in processing large amounts of data and capable of recognizing patterns that might not be visible to humans or simpler models (Shahin et al., 2025). However, implementing this method is more challenging because it requires advanced technology, not only for developing the program but also for its operation. As a result, many companies have limited access to this method (Ferreira et al., 2025). Furthermore, the complex and non-transparent decision-making process is often seen as a black box in this model (Shahin et al., 2025).

RQ2: In which operational conditions are AI methods proven to outperform traditional statistical methods, and vice versa?

From 28 selected articles that apply statistical and AI methods in inventory management, we classify the role of these methods in decision support complexity. Decision support refers to whether the proposed method is limited to generating forecasting outputs for managers to make decisions or if it can also produce operational inventory decisions, such as the quantity to order, the right time to order, reorder levels, and so on. From Table 7, it can be seen that 50% of articles use methods with high decision-support complexity to assist inventory management in the manufacturing industry.

**Table 7:** Comparative overview of statistical and AI methods for inventory management in the manufacturing industry.

References	Methods	Algoritm	Decision support complexity	Accuracy (%)	Reported effectiveness
Statistical method					
Affonso et al. (2024)	Heuristic method	Bootstrap WSS, B2-OPT, BSWAP	High	89%	Suitable for demands with higher variance
(Aiello et al. 2025)	Optimization method	CLSC	Very High	Not mention	Effective for analyzing different configurations
(Fattah et al. 2018)	Time series analysis	ARIMA	Medium	Based AIC/SBC	Large and consistent historical data is available
(Karpagavigneswari and Kamali 2025)	Optimization method	Red Panda Optimizer with Logistic Mapping	High	94%	Useful in dynamic and volatile environments
(Kourentzes and Trapero 2020)	Time series analysis	Level exponential Smoothing	High	Reduction bias 62.4%	Helpful when transforming estimates into complex decisions
(Kumar et al. 2024)	Time series analysis	SES	Very High	87%	Effective for inventory management of perishable products
(Li et al. 2023)	Time series analysis	FIDE, DIVIDE	Medium	79%	Suitable for intermittent demand
(Tliche and Taghipour 2020)	Time series analysis	WMA and Newton	High	Reduction error 19.6%	Effective for higher parameter values than lead time
Artificial intelligence					
(Ahakonye et al. 2024)	Deep learning	LightGBM	High	50.99%	Suitable for large datasets
(Arvind et al. 2025)	Supervised learning	XGboost, RF classifier	Very High	XGBoost: 95%, RF: 89%	Effective in dynamic and fast paced environments
Bo and Xu (2024)	Optimization algorithm	PSO	High	85%	Handles large volumes of data and adapts quickly to changes
(Danach and Dirani 2024)	Supervised learning	Decision tree, RF, gradient boosting	High	DT: 82%, RF: 88%, GB: 90%	Effective for forecasting with many variables
(Hajek and Abedin 2020)	Supervised learning	RF, k-Nearest Neighbor, Decision Tree, SVM, and NN	Very High	91.57%	Effective for imbalanced big data
(Jackson and Jesus Saenz 2024)	Natural language processing	GPT 3 -Codex	High	Not mention	Generates simulations using verbal descriptions
(Jaipuria and Mahapatra 2019)	Genetic programming	DWT-MGGP	High	97.67%	Practical for nonlinear and non stationary demand
(Jauhar et al. 2024)	Heuristic	NCAI	Medium	81.43%	Handles incomplete data
(Liu et al. 2025)	Reinforcement learning	HAPPO	Very High	Not mention	Effective with limited and complex information
(Pasupuleti et al. 2024)	Supervised learning	LSTM, CNNs	High	95%	Ideal for large data sets
(Preil and Krapp 2022)	Reinforcement learning	Monte Carlo Tree Search (MCTS),	High	Not mention	Capable of managing complex and uncertain large scale data
(Raouf et al. 2025)	Supervised learning	AI based optimization framework	High	84.31% stock optimization	For companies operating under a Just-In-Time (JIT) system
(Salinas et al. 2020)	Deep learning	RNN	Medium	42% reduction error	For millions of data points
(Sandra et al. 2025)	Supervised learning	Fuzzy multi criteria	Very High	Not mention	Effective for dynamic and uncertain data
(Shahin et al. 2025)	Supervised learning	CART, ELM, GPR, KNN, MLP, SVR, XGB, LSTM	Low	• CART: 90.91% • ELM: 96.29 % • GPR: 99.47 % • KNN: 87.22 % • MLP: 98.32 % • SVR: 95.24 % • XGB: 90.3 % • FBP: 97.16 % • LSTM: 98.67 %	Suitable for data with complex and nonlinear patterns, large datasets with multiple features, and changing trends and seasonality
	Deep learning	BNN, FCNs, GRNN, RNN, TFT	Low	• BNN: 93.41 % • FCN: 89.46 % • GRNN: 79.4 % • RNN: 99.55 % • TFT: 90.22 %	
(Singh and Mishra 2024a)	Supervised learning	Decision Tree Classifier	High	Not mention	Effective when product demand varies seasonally
(Singh and Mishra 2024b)	Supervised learning	Decision Tree Classifier	High	Not mention	Effective for products with seasonal demand
(Spiliotis et al. 2022)	Deep learning	MLP, BNN	Low	MLP: 91.3 % BNN: 89.1 %	For daily demand
	Supervised learning	RF, GBT, K-Nearest, SVR, GP	Low	RF: 96.8 % GBT: 97.5 % KNNR: 96.5 % SVR: 96.5 % GP: 93.2 %	For sparse and noisy data.
(Sukolkit et al. 2024)	Supervised learning	LSTM (CNN, RNN, GRU)	High	98.35%	Excels at capturing trends, seasonal patterns, missing data, and holiday effects
(Wahedi et al. 2023)	Deep learning	ANN	Medium	65.42 %	Suitable for varying demand

**Figure 8:**
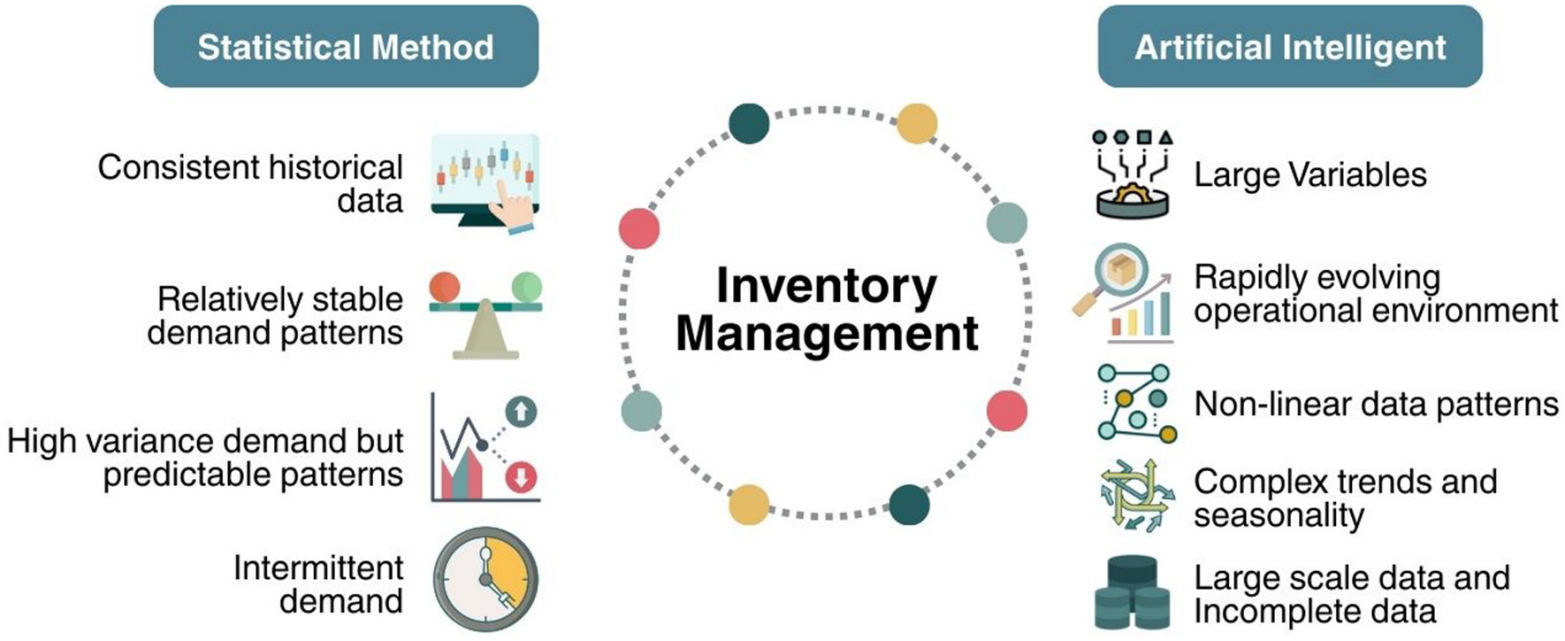
Comparison of conditions for applying statistical and AI methods in inventory management.

Before conduct the analysis, it is important to acknowledge that the 32 included studies are methodologically heterogeneous. They differ in data types, forecasting horizons, evaluation metrics (accuracy, RMSE, MAE, MAPE, cost reduction, etc.), and experimental designs. Therefore, the comparative observations presented in this section are based on directional trends reported within each study's own experimental context, rather than a standardized cross-study benchmark. Readers should interpret these comparisons as qualitative patterns, not as equivalent numerical benchmarks. When we refer to articles that apply statistical methods in inventory management, the highest accuracy reported is only 94% as reported within its own experimental setting and dataset (Karpagavigneswari and Kamali, 2025). Meanwhile, AI technology with accuracy exceeding 95% has reached nearly 32% (Arvind et al., 2025; Jaipuria and Mahapatra, 2019; Pasupuleti et al., 2024; Shahin et al., 2025; Spiliotis et al., 2022; Sukolkit et al., 2024). This shows that AI methods possess an advantage in terms of accuracy. However, it should be noted that developing AI for inventory management requires significant initial investment costs. Additionally, managers and operational staff must be trained to carry it out. Therefore, basic statistical methods can still be applied in inventory management even with limited resources. Figure 8 shows the comparison of conditions for applying statistical and AI methods in inventory management.

Statistical methods have shown to be highly effective for inventory management when operational conditions align with consistent historical data (Fattah et al., 2018), relatively consistent demand patterns (Kumar et al., 2024), high variance demand with predictable patterns (Affonso et al., 2024), and even capable of predicting demand for goods that do not occur in every period, known as intermittent demand (Li et al., 2023; Spiliotis et al., 2022). Some statistical methods used not only forecast but also assist decision makers in making more effective and efficient inventory decisions. These include the heuristic method (Affonso et al., 2024), CLSC (Aiello et al., 2025), Red Panda Optimizer (Karpagavigneswari and Kamali, 2025), Exponential Smoothing (Kourentzes and Trapero, 2020; Kumar et al., 2024), and Weighted Moving Average (WMA) combined with optimization using Newton (Tliche and Taghipour, 2020). The CLSC model aims to maximize profits by evaluating the value and cost of sales along with remaining inventory that can be reused or recycled (Aiello et al., 2025). To generate decision support, the methods used need to be integrated with various approaches, as demonstrated by Kumar et al. (2024) by integrating Simple Exponential Smoothing (SES) directly into the dynamic pricing and inventory control system.

Research conducted by Jahin et al. (2024) also states that simple statistical methods tend to be ineffective for large datasets or those with overlapping classes. However, statistical methods have a transparent process, which allows them to support managerial decisions that require justification (Jahin et al., 2024). Furthermore, research Oikonomou and Damigos (2025) comparing the application of Machine Learning methods, especially LightGBM and ARIMA, for forecasting base metal prices indicates that ARIMA achieves lower RMSE values in estimating tin and lead prices than LightGBM. This indicates that, in specific situations, traditional statistical approaches are still preferable, and more complex models do not always lead to improved forecasts (Oikonomou and Damigos, 2025).

Although statistical methods have proven effective in various conditions, AI methods can achieve higher accuracy and reduce errors and costs. According to Xu and Bo (2025), the application of AI can reduce total supply chain costs by 12% and improve resource utilization efficiency by up to 20%. Additionally, AI methods show significant advantages in more complex conditions (Shahin et al., 2025). This can be seen from the use of AI models such as LightGBM, XGBoost, random forest, and gradient boosting, which are effective when there are many variables and the operating environment changes rapidly (Ahakonye et al., 2024; Arvind et al., 2025; Danach and Dirani, 2024). Furthermore, AI methods have demonstrated their superiority in situations involving nonlinear patterns, complex trends, seasonality, large-scale data, or incomplete datasets, as they can learn intricate patterns that simple statistical methods cannot capture (Jaipuria and Mahapatra, 2019; Liu et al., 2025; Pasupuleti et al., 2024; Preil and Krapp, 2022; Sukolkit et al., 2024).

This aligns with a study by Kang et al. (2025), which found that machine learning methods are used when ARIMA statistical methods are limited in capturing the complexity of demand patterns in volatile business environments. In highly volatile sectors, demand is unpredictable, so ARIMA models that rely solely on temporal autocorrelation patterns cannot capture sudden demand spikes. Thus, AI methods are an effective solution for handling complex supply chain issues and supporting more efficient decision-making and resource allocation (Mao, 2025).

RQ 3: How extensively does existing literature explore hybrid approaches that combine statistics and AI? What are the advantages of these methods, and what challenges do they encounter?

According to the findings of researchers, literature on the application of hybrid methods, specifically combining statistical and AI methods into a single system, remains somewhat limited, particularly in inventory management within the manufacturing industry. Of the selected articles, only four used hybrid methods in inventory management (Table 8). In these articles, statistical and AI methods were used within a single inventory management system but served different and complementary tasks. The four articles emphasized that AI methods alone are not sufficient to be used independently. The reviewed hybrid studies suggest that combining statistical and AI components may improve inventory decision support in specific contexts, but the evidence remains limited and does not justify broad claims of superiority. The most widely used AI method was deep learning (37.5%). This method was integrated with nonparametric methods and time series analysis (Gammelli et al., 2022; Kim et al., 2022; Sagaert and Kourentzes, 2024). Given that only four studies within the final sample satisfied the review criteria for a hybrid approach within a single inventory decision framework, the findings presented in this section should be regarded as exploratory and illustrative rather than conclusive.

**Table 8:** An overview of hybrid method.

References	Methods	Algoritm	Task	Limitation
Gammelli et al. (2022)	Deep learning	Variational Poisson Recurrent Neural Network (VP-RNN)	To predict a statistical inventory optimization	Limited External Variables
	Non-parametric	User Dissatisfaction Function (UDF)	To determine daily starting inventory levels with various forecasting methods	Misalignment of Prediction and Decision Objectives
Kim et al. (2022)	Deep learning	LSTM	To predict point estimation with historical data	Incomplete data leads to inaccurate estimates
	Non-parametric	2D kernel density estimation (KDE)	To predict interval estimation with kernel density estimation	Limited integration of the proposed algorithm
Kumar et al. (2024)	Reinforcement Learning	Genetic Algorithms	To find optimal solutions for complex problems	Difficulty in unifying real time data streams
	Time Series Analysis	ARIMA	To forecast seasonal and irregular data	Limited data quality and high computational costs
Sagaert and Kourentzes (2024)	Deep learning	LightGBM	To leverage leading indicators for improved aggregate forecasting	Limited leading indicators
	Time Series Analysis	Exponential Smoothing, Lasso Regression	To generate univariate forecasts at the disaggregate level	Insensitive to seasonal data

The four selected hybrid studies reveal three recurring design principles that indicate a recurring hybrid design pattern in manufacturing inventory management. First, the AI layer is consistently assigned to handle complex, nonlinear, and data-intensive tasks, such as capturing temporal demand patterns, dynamic pricing, and adaptive market responses, where statistical models are insufficient. Second, the statistical layer handles structured tasks where interpretability, uncertainty quantification, and low computational cost are paramount, including interval estimation, variable selection, and time-series decomposition. Third, a decision integration mechanism connects both layers, translating forecast outputs into actionable inventory parameters such as reorder points, safety stock levels, and optimal order quantities.

This modular design is most explicitly illustrated by Kim et al. (2022), where LSTM is employed for point prediction of stochastic demand, while 2D Kernel Density Estimation (KDE), a non-parametric statistical technique, models the variability of that prediction to generate reliable cost-weighted prediction intervals. The statistical layer does not replace the AI forecast but extends it into an operationally useful range, enabling small and medium-sized manufacturers with volatile and sparse data to determine inventory levels that minimize both understock and overstock costs.

Sagaert and Kourentzes (2024) illustrate a hierarchically organized hybrid model, where LightGBM identifies macroeconomic leading indicators at the aggregate demand level. Meanwhile, Exponential Smoothing and LASSO regression produce interpretable and scalable forecasts at the individual Stock Keeping Unit (SKU) level. These statistical techniques are applied in contexts where their strengths are greatest at disaggregate levels characterized by high noise, small sample sizes, and a need for interpretability for management purposes. Conversely, the AI methodology operates most effectively at the aggregate level, where complex, nonlinear interactions among market indicators and demand exist. Forecast reconciliation via hierarchical methods then transfers the leading indicator information from the aggregate level down to the SKU level which actual inventory decisions are made.

Gammelli et al. (2022) analyzed that the deep learning method Variational Poisson Recurrent Neural Network (VP-RNN) can also be integrated with the statistical method User Dissatisfaction Function (UDF). Combining these methods can determine optimal starting inventory levels. This study exposes a critical design insight for hybrid frameworks, predictive accuracy and prescriptive (inventory decision) accuracy are not always aligned. A model with higher individual forecast accuracy does not necessarily yield better inventory decisions, because operational outcomes depend on the joint behavior across all demand streams and their interaction with the inventory policy. This finding has direct implications for how hybrid models should be evaluated, not exclusively by forecast error metrics (e.g., RMSE, MAE) but also by downstream decision performance such as fill rates, stockout frequency, and total holding cost (Gammelli et al., 2022).

Kumar et al. (2024) present the most technically integrated hybrid model, combining Reinforcement Learning (RL) and Genetic Algorithm (GA) for adaptive pricing and multi-echelon replenishment optimization. This approach employs Kalman filtering and exponential smoothing for stochastic demand forecasting. In this framework, the statistical layer continuously updates demand estimates via a Bayesian mechanism that adjusts predictions based on observed deviations, thus providing a stable and interpretable baseline. The AI-RL layer then operates upon this baseline to dynamically modify prices and replenishment quantities in real-time, considering deterioration rates, blockchain-verified supplier data, and carbon emission constraints. The modular design allows the statistical forecasting module to be decoupled and replaced independently of the RL optimization layer, and vice versa. This architecture underscores the efficacy of hybrid systems within complex, multi-objective supply chains. Among the four selected articles, it was found that AI methods are used to handle complex and nonlinear predictive tasks, especially in capturing temporal patterns, nonlinear interactions, and adaptive market dynamics. Conversely, statistical methods play a crucial role in modeling uncertainty, enhancing transparency, and improving decision-making with deep interpretability (Gammelli et al., 2022; Kim et al., 2022; Kumar et al., 2024; Sagaert and Kourentzes, 2024).

The findings of Gonçalves et al. (2021) state that statistics and AI can be used simultaneously. The ARIMAX statistical model is more suited for the early stages of the life cycle of a product because of limited historical data, whereas machine learning methods are applied throughout the entire life cycle of components, with their effectiveness increasing as more historical data is available Gonçalves et al. (2021). This aligns with the findings of Zietsman and Van Vuuren (2023), that statistical methods are used to provide an interpretable and reliable foundation for forecasting and anomaly detection, while AI methods are responsible for managing data complexity and advanced pattern recognition. Combining these two methods will greatly support a dynamic, multi-stage, decision support-oriented inventory system (Zietsman and Van Vuuren, 2023). Although the hybrid approach offers significant technical advantages, it also encounters limitations and challenges. Hybrid methods that yield more accurate predictions do not always lead to better inventory decisions (Gammelli et al., 2022). Furthermore, the increasing complexity of the model will complicate implementation in industrial environments with limited resources in real industries (Kim et al., 2022).

Taken together, these findings suggest that the contribution of this review extends beyond merely describing publication trends. The review systematically synthesizes how various methodological approaches serve distinct roles in manufacturing inventory management, the operational conditions under which they are most appropriate, and why hybridization remains a promising yet underdeveloped area. In this context, the study provides a structured explanation of method suitability and underscores the necessity of evaluating forecasting techniques not solely based on predictive accuracy but also considering their downstream implications for inventory decision-making.

RQ4: What are the technical challenges, limitations, and recommendations for future research on optimizing the integration of statistical methods and AI in inventory management decision- making?

The application of statistical integration and AI in inventory management within the manufacturing industry encounters several significant technical challenges. A primary obstacle is the limited availability of long-term, high-quality historical data in the industry (Bo and Xu, 2024). This is because making accurate inventory decisions depends on high-quality data that is available in real time. On the other hand, current literature reviews have several limitations, such as 34.38% of the studies using simulated data or synthetically generated data rather than empirical data from real manufacturing environments, so validation for application in actual manufacturing industry conditions is limited. While simulation serves a legitimate purpose in exploring theoretical model behavior under controlled conditions, it carries fundamental limitations when applied to real-world manufacturing contexts. Simulated data typically assumes clean, complete, and stationary distributions, whereas actual manufacturing environments are characterized by noisy data from sensor errors and manual entry mistakes, missing values due to system downtime or recording failures, irregular demand driven by unplanned disruptions such as machine breakdowns or supplier delays, and non-stationarity caused by seasonal shifts, price changes, or market shocks. Models trained and validated exclusively on simulated data may therefore exhibit artificially inflated performance metrics that do not generalize to real operational conditions. This gap between simulation performance and real-world applicability is a recognized threat to the external validity of the findings reported in this literature. The second limitation is the lack of a standardized hybrid framework that is easy to adapt across different manufacturing industries. Finally, the evaluation remains too narrowly focused on forecasting accuracy alone, without considering the broader impact of inventory decisions.

Based on the 32 selected papers, research on the application of statistics and artificial intelligence in inventory management remains limited in terms of accuracy. Among the reviewed studies, only a minority explicitly connect forecasting outcomes to subsequent inventory decisions, such as safety stock levels, reorder points, or service level objectives. This indicates that the field has not yet systematically investigated how statistical and AI-driven forecasting methods are translated into practical inventory management strategies. This observation constitutes a significant contribution of this review, as it identifies the current knowledge gaps and delineates a clear trajectory for future research. Future field research should move beyond just comparing forecast accuracy and focus more on methods that directly aid inventory decisions in manufacturing contexts. In particular, more research should explore hybrid approaches that combine the interpretability of statistical models with the adaptability of AI because the current evidence indicates that hybrid systems are promising but still underdeveloped in the literature. Greater focus should also be placed on interpretable machine learning, probabilistic forecasting, and prescriptive AI methods that can convert demand predictions into operational decisions such as safety stock levels, reorder points, replenishment timing, and multi-echelon coordination. For relatively stable and data-limited settings, future studies should continue to improve statistical-optimization methods, especially for intermittent demand and situations where managerial explainability is crucial. In contrast, in highly dynamic and data-rich environments, more research is needed on advanced supervised learning, deep learning, and reinforcement learning techniques, especially when demand patterns are nonlinear, rapidly changing, and affected by multiple internal and external factors variables. Additionally, more empirical research using real industry data is necessary, as many current studies still depend on simulated data. The most valuable datasets include historical sales and inventory records, along with lead times, stockout events, replenishment cycles, pricing, promotions, supplier performance, production constraints, and other contextual variables that shape demand and inventory decisions. More SKU-level, warehouse-level, and multi-echelon datasets at the SKU and warehouse levels, observed over extended periods, are necessary for researchers to assess forecast accuracy, inventory costs, service levels, overstocking, stockouts, and resilience outcomes. Lastly, interdisciplinary collaboration between scientists and industry practitioners is needed to develop inventory management methods that can be effectively applied in real manufacturing industries.

Overall, the answers to RQ1–RQ4 indicate that research on inventory management in manufacturing has shifted from mainly statistical forecasting methods to a wider range of techniques, including supervised learning, deep learning, reinforcement learning, and emerging hybrid systems. However, this change should not be interpreted as a simple linear transition from traditional to advanced methods, as this review consistently indicates that the suitability of a method still depends on demand patterns, data availability, interpretation needs, and the specific inventory decisions being made. RQ1 identified the growing variety of methods, RQ2 pointed out the contextual factors influencing method performance, RQ3 showed the potential but still limited development of hybrid approaches, and RQ4 exposed ongoing technical and empirical limitations that restrict progress in the field.

## 5 Implications, limitations, and future research about inventory management

This study offers both academic and practical implications for inventory management research and practice. This study contributes to theory and analyzes the latest developments in the application of statistics and AI to decision-making in inventory management. In this regard, the most frequently used statistical techniques, AI, and hybrid methods in inventory management research are discussed to provide a comprehensive overview of the current literature. The last RQ highlights the limitations of current research involving statistics and AI in inventory management, offering insights for future studies in this field. Our findings also offer valuable insights for managers and practitioners. The integration of statistics and AI is crucial to enhancing the effectiveness and efficiency of inventory planning in the manufacturing industry. Excess stock can increase operational costs, including higher warehouse expenses, decreased company cash flow from inventory purchases, and the risk of stock damage if it does not sell (Jauhar et al., 2024). Conversely, a lack of stock will lead to delays in fulfilling demand. These different statistical and AI techniques play a crucial role in enhancing inventory management performance in the manufacturing industry (Xiao et al., 2024). These findings indicate that hybrid approaches constitute a promising avenue for enhancing inventory management within the manufacturing industry. However, the existing evidence base remains insufficient to definitively assert that they represent the optimal solution across varying contexts. From the perspective of a practitioner, this research can serve as a guide for manufacturing companies to gradually implement technology. Companies need to consider their level of organizational maturity, infrastructure constraints, quality of historical data, skills of available human resources, and the culture of innovation within the organization. Ultimately, by integrating statistics and AI, companies can reduce inventory costs due to overstocking or stockouts, become more responsive to demand fluctuations, and maintain sustainable competitiveness in this global era.

Like any literature review, this study has certain limitations that also suggest directions for future review research. First, the review is limited to inventory management in the manufacturing industry, which restricts the generalizability of the findings to other sectors with different demand structures, such as retail, healthcare, spare parts, and e-commerce. Second, although this review examines various statistical, AI, and hybrid methods, most of the studies collected and analyzed focus on improving forecasting accuracy, while the assessment of their direct impact on operational inventory decisions, such as safety stock and reorder points, remains limited. Third, the number of studies that actually implement a hybrid approach combining statistics and AI within a single inventory decision framework remains very limited, so conclusions about the effectiveness of this hybrid method remain exploratory and lack extensive empirical evidence.

Based on these limitations, future systematic reviews should compare evidence across sectors to determine whether the relative performance of statistical, AI, and hybrid methods varies under different levels of volatility, intermittency, perishability, and product life-cycle uncertainty. This expansion is crucial to understanding how the performance of this hybrid method between statistics and AI adapts to sectors with high volatility, such as retail or e-commerce. Additionally, Future reviews should also expand the publication window and include more recent studies in order to capture the rapid development of AI-based and hybrid methods. In addition, future review articles should examine more explicitly how hybrid systems are structured, which components are statistical vs. AI-based, how these components interact, and whether hybridization improves not only predictive performance but also managerial interpretability and implementation feasibility.

Another limitation of this paper relates to the scope of database coverage. This study exclusively utilizes the Scopus database. Although Scopus offers a comprehensive selection of multidisciplinary journals, incorporating the Web of Science as an additional source may help prevent the omission of potentially pertinent studies. Consequently, future research should consider including other databases as sources for article retrieval.

## 6 Conclusion

This paper review thoroughly examines the role of statistical methods and AI in management decision-making within the manufacturing industry. This paper employs the PRISMA method to provide a well-structured framework for conducting systematic reviews and meta-analyses, enabling a comprehensive exploration of the topic. This paper review shows that research on inventory management in manufacturing has shifted from predominantly statistical approaches toward a broader methodological landscape that increasingly includes AI and emerging hybrid systems. Statistical methods remain valuable in contexts characterized by stable demand, consistent historical data, and strong needs for interpretability, whereas AI methods tend to perform better in dynamic environments with large-scale, nonlinear, and uncertain demand patterns. At the same time, the review indicates that more advanced methods do not automatically guarantee superior practical performance, because method suitability depends on data characteristics, operational context, and the specific inventory decision being supported. The findings also suggest that hybrid approaches are a promising but still underdeveloped direction for manufacturing inventory management. Because the evidence is drawn from only a small number of studies, conclusions regarding their effectiveness should be treated with caution. Therefore, future research should place greater emphasis on real industrial data, decision-relevant performance measures, interpretable AI, and more structured hybrid frameworks that can be implemented effectively in actual manufacturing settings.

## Data Availability

The original contributions presented in the study are included in the article/supplementary material, further inquiries can be directed to the corresponding author.
